# Supplementary material for: MetaRibo-Seq measures translation in microbiomes
Source: Nat Commun. 2020 Jun 29;11:3268. doi: 10.1038/s41467-020-17081-z (PMC7324362; doi:10.1038/s41467-020-17081-z)
Supplement: Supplementary file 10 — Supplementary Data 7 [file 41467_2020_17081_MOESM10_ESM.zip › File2/Confidence_High_Taxonomy/292093_out.krona.html]

Javascript must be enabled to view this page.

members
magnitude
magnitudeUnassigned
count
unassigned
taxon
rank

292093\_out

1

SRS023914\_contig\_number\_29119
5

1
3
superkingdom

SRS051031\_contig\_number\_36069
2


SRS013687\_contig\_number\_9790SRS1041145\_contig\_number\_31450
976
phylum
2


SRS098571\_contig\_number\_contig-100\_2414.294611
1
